# Meeting of the Chicago Microscopical Club—Structure of the Blood Corpuscles

**Published:** 1869-02

**Authors:** 


### MEETING OF THE CHICAGO MICROSCOPICAL CLUB—STRUCTURE OF THE BLOOD CORPUSCLES.

The Chicago Microscopical Club held an adjourned meeting last evening, at Rush Medical College, by invitation of Prof. J. W. Freer, M. D., to witness his exposition of the anatomy of the blood cells.

At the regular meeting of the Club held at the Academy of Sciences, January 26, W. W. Allport, D. D. S., was elected President, James Hankey, Vice President, Henry F. Munroe, Esq., Treasurer.

In stating the object of the meeting the President said that the existence of a nucleus in blood corpuscles has been long a question with histologists. Such distinguished men as Drs. Carpenter, Dalton, Peasley, Wharton, Jones, Kolliker, Bennett, Beale and McDonald, have denied their existence. Virchow is the only author who has strongly inclined to the opinion of their existence, and he simply assumes the fact without clearly demonstrating it. Within the last twelve months Prof. Freer, of this city, has discovered in these corpuscles characters which have never before been mentioned by any author on human histology. In his recent visit to Europe he exhibited their structure to Prof. Hughes Bennett, of Edinburgh, who subsequently stated to a physician of this city that Prof. Freer had presented to him characters in the blood cells which he had never before witnessed. Other prominent European histologists made the same frank acknowledgment. If what Dr. Freer should exhibit shall prove to be the real nuclei of the blood cells, it will be sufficient to hand his name down in the history of medicine as the discoverer of what has long been sought, but never before found, and will entitle him to the gratitude of all lovers of science.

Prof. Freer, in presenting the subject of the evening, said that histologists acknowledge the existence of a nucleus in the reptitian blood cells. He had always believed the blood cells of warm blooded animals nucleated also. When viewed by transmitted light the structure is lost, and it is only when shown by reflected light as an opaque object—which he was able to do by the use of an illuminator invented and patented by Prof. H. L. Smith, of Geneva, N. Y.—that the anatomical structure of the cell is truly exhibited. This shows the cell as a bi-concave disc with the nucleus appearing as a prominence in the center. He did not make this as a positive assertion, but exhibited the object and left scientific men to draw their own inferences.

Dr. S. J. Jones stated that he saw Prof. Bennett subsequently to his meeting with Prof. Freer, and that Prof. Bennett expressed his high satisfaction with the presentation of the cells which Prof. Freer made.

The Club then proceeded to inspect the corpuscles, and, subsequently, other interesting preparations made by Dr. W. C. Hunt.

After a brief discussion of the points presented, and the adoption of a vote of thanks to Prof. Freer and Dr. Hunt for the gratification received, the Club adjourned.

